# Plasma total and donor-derived cell-free DNA predict survival in kidney transplant recipients

**DOI:** 10.3389/frtra.2025.1624291

**Published:** 2025-09-01

**Authors:** Alison S. Graver, David A. Power, John B. Whitlam

**Affiliations:** ^1^ Department of Medicine, University of Melbourne, Parkville, VIC, Australia; ^2^ Department of Nephrology, Austin Health, Heidelberg, VIC, Australia; ^3^ Australian Centre for Transplantation Excellence and Research, Austin Health, Heidelberg, VIC, Australia; ^4^ Victorian Clinical Genetics Services, Murdoch Children’s Research Institute, Parkville, VIC, Australia

**Keywords:** kidney transplantation, ddcfDNA, cell-free DNA—cfDNA, prognostic biomarker, survival

## Abstract

**Introduction:**

Studies evaluating cell-free DNA (cfDNA) in kidney allograft dysfunction have primarily focused on detection of rejection by donor-derived cfDNA (ddcfDNA). The utility of ddcfDNA as a marker of longer-term outcomes has not been examined.

**Methods:**

This study investigated the prognostic value of plasma total cfDNA, fractional ddcfDNA and absolute ddcfDNA, quantified in 49 adult kidney transplant recipients (KTRs) at the time of indication allograft biopsy between 2014 and 2017. Primary outcomes were death, death-censored graft loss (DCGL), and all graft loss (AGL).

**Results:**

During a median follow-up of 6.3 years, 7 patients died, 7 experienced DCGL, and 14 had AGL. Death was predicted by high total cfDNA [>4,034 copies/ml, hazard ratio (HR) 5.94, 95% CI 1.40–25.13, *P* = 0.008] and low fractional ddcfDNA (<0.67%, HR 10.85, 95% CI 1.32–1,408.19, *P* = 0.03), and DCGL was predicted by high fractional ddcfDNA (>0.72%, HR 4.93, 95% CI 1.12–21.72, *P* = 0.04), on univariate analysis. AGL was predicted by high total cfDNA (>4,034 copies/ml, HR 642, 95% CI 1.15–3.56 × 10^5^, *P* = 0.045) on multivariate analysis. Absolute ddcfDNA was not associated with survival outcomes.

**Discussion:**

This study demonstrates potential prognostic utility of total cfDNA and fractional ddcfDNA in KTRs with allograft dysfunction. Incorporation of these biomarkers could enhance personalised care, beyond non-invasive detection of rejection.

## 1 Introduction

Studies concerning cell-free DNA (cfDNA) in kidney allograft dysfunction have primarily focused on the performance of donor-derived cfDNA (ddcfDNA) to detect prevalent rejection. The utility of ddcfDNA as a longer-term prognostic marker has not been examined in kidney transplant recipients (KTRs).

Higher plasma ddcfDNA levels increase the likelihood of biopsy-proven allograft rejection, and significantly elevated ddcfDNA levels strongly predict the presence of antibody-mediated rejection (AMR) (1). Emerging evidence also suggests an association between elevated ddcfDNA and *de novo* donor-specific antibody (DSA) (2). As AMR and *de novo* DSA are predictors of long-term allograft outcomes, we hypothesised that higher levels of ddcfDNA would associate with poorer allograft outcomes.

In addition to quantifying fractional ddcfDNA as a percentage of total cfDNA, our approach measured the absolute concentrations of ddcfDNA and total cfDNA in copies per millilitre of plasma. Studies of total cfDNA in non-transplant recipients have demonstrated elevation in many physiological and pathological states, such as pregnancy, critical illness, sepsis, myocardial infarction, and stroke (1). There is limited data in recipients of solid organ transplants (SOTs). Extreme elevations have been observed in KTRs with inflammatory illnesses including COVID-19 (3), bacteraemia, cytomegalovirus (CMV) infection and haemoptysis (4). As cardiovascular disease and infection are major causes of death in transplant recipients, we further hypothesised that higher total cfDNA levels would associate with poorer patient outcomes in KTRs.

In this study, we aimed to evaluate the long-term prognostic value of plasma absolute and fractional ddcfDNA and total cfDNA in the cohort used for diagnostic validation of our ddcfDNA quantification method (4). In the original study, participants underwent ddcfDNA and total cfDNA assessment immediately before indication kidney transplant biopsy, providing a well-characterised cohort for assessing long-term outcomes.

## 2 Patients and methods

### 2.1 Population and data collection

**Figure 1:**
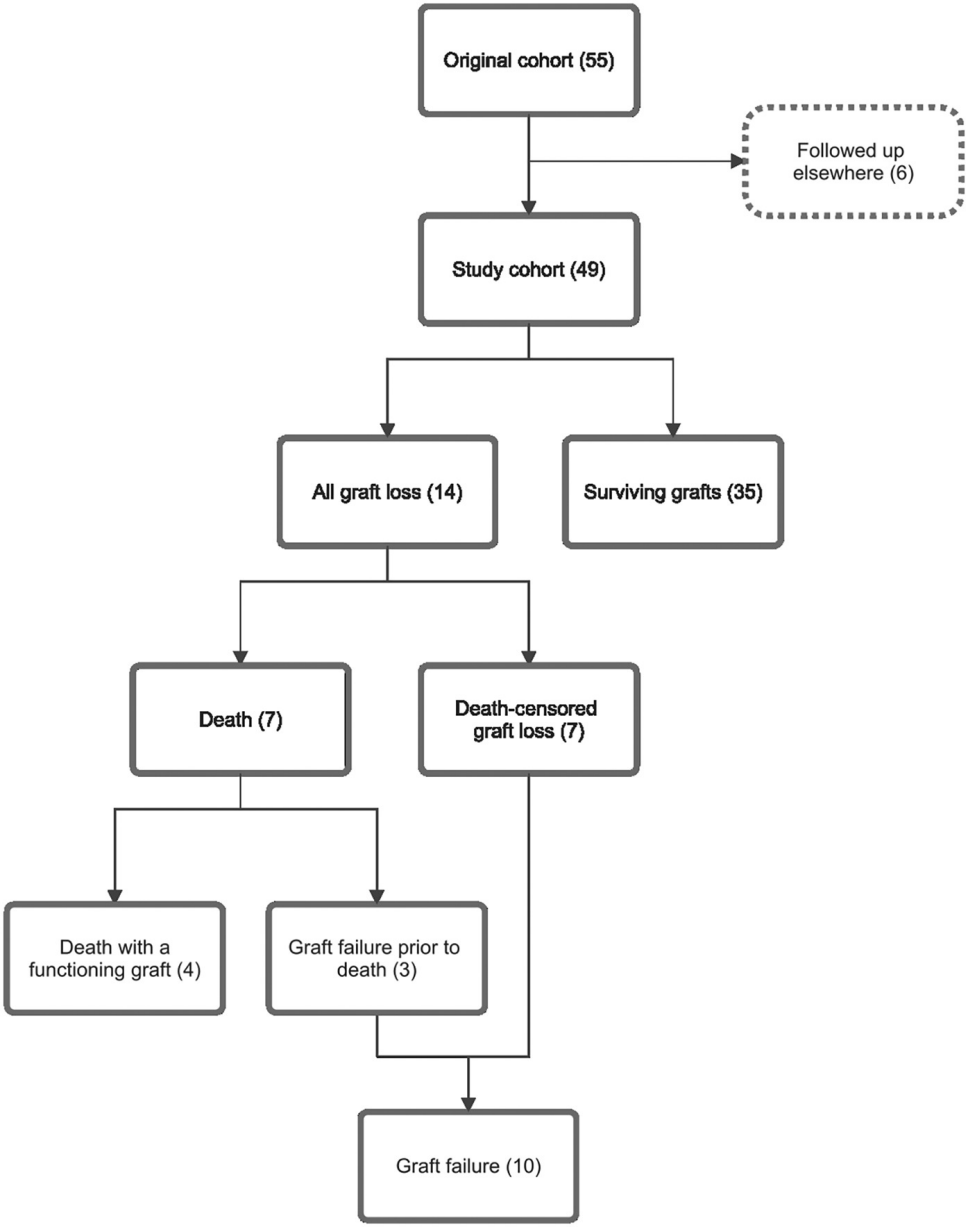
Study cohort flow diagram and relationships between outcome subgroups. This outcome analysis included 49 of 55 participants in the original cohort; six participants were excluded as their follow up occurred elsewhere. Fourteen participants experienced allograft loss, including 4 deaths with functioning grafts, 3 deaths following allograft failure, and 7 participants with death-censored graft loss. Numbers in brackets represent number of cases.

The original cohort included adult kidney transplant recipients who underwent allograft biopsy to evaluate graft dysfunction, indicated by a rise in creatinine or worsening proteinuria, between 2014 and 2017. Singular cfDNA measurements were performed on plasma samples collected at the time of biopsy. cfDNA quantification and histopathological analysis were described in the original paper (4). Absolute cfDNA and ddcfDNA concentrations are presented as “one-copy” equivalents per millilitre of plasma (cp/ml). Recipients who did not receive transplant care at the primary institution were excluded from this outcome analysis due to insufficient follow-up data (Figure 1). Clinical information was retrospectively collected from the electronic medical record. The study was approved by the institutional human research ethics committee.

### 2.2 Outcome measures

Primary outcomes were death, death-censored graft loss (DCGL, defined as living participants with failed grafts who had recommenced dialysis), and all graft loss (AGL, encompassing participants who died or experienced DCGL) (Figure 1). Secondary outcomes included death with a functioning graft, graft failure (defined as all participants who resumed dialysis, regardless of subsequent death), and graft nephrectomy.

The study period began on the date of cfDNA assessment and continued until a censoring event occurred. Participants had follow-up until administrative censoring on 31 March 2022.

### 2.3 Covariates

Potential covariates analysed included age at the time of cfDNA assessment, sex, primary kidney disease, transplant vintage (years from transplantation to cfDNA assessment), kidney donor age, donor category, donor HLA antigen mismatch, pre-existing DSA, serum creatinine (µmol/L), urine protein-to-creatinine ratio (PCR, mg/mmol), urine albumin-to-creatinine ratio (ACR, mg/mmol), urine white cell count (WCC, ×10^6^/L), urine red cell count (RCC, ×10^6^/L), plasma WCC (x10^9^/L), plasma lymphocyte count (x10^9^/L), plasma neutrophil count (x10^9^/L), CMV DNAemia, BK viraemia, DSA detection, rejection episodes, and immunosuppression. Time-varying covariates were recorded at 0, 1, 2, 4, and 5 years, as well as at the censoring or end date.

### 2.4 Statistical analysis

Baseline characteristics of the study cohort were expressed as mean with SD, or median with interquartile range (IQR), depending on the data distribution. Groups were compared using the Mann–Whitney *U* test for non-parametric variables. Correlations between continuous variables were assessed using Pearson's product-moment correlation.

For survival analyses, optimal cut points for each cfDNA parameter were determined by maximally selected rank statistics to account for differences in follow-up times across participants (5, 6). Total cfDNA and ddcfDNA (absolute and fractional values) were classified as “low” or “high” based on these cut points. Kaplan–Meier survival curves were generated for each cfDNA parameter and stratified by these classifications. Statistical differences between groups were evaluated using log-rank tests. Hazard ratios (HRs) for outcomes were estimated using standard Cox proportional hazards models. In the event of non-proportional hazards, weighted Cox regression models were used to determine average HRs and generalised concordance probabilities (7).

Two multivariate Cox proportional hazards regression models were constructed for each primary outcome to evaluate the predictive value of total cfDNA and ddcfDNA. Model 1 included clinically relevant covariates: age, transplant vintage, donor category, HLA mismatch, and any rejection episode during follow-up. For AGL and DCGL outcomes, pre-existing DSA was additionally included as a covariate. Model 2 adjusted for covariates that were statistically significant (*P* < 0.10) in univariate analyses. Participants were stratified according to the cfDNA and ddcfDNA cut points specific to each outcome.

The significance of individual predictors in multivariate models was assessed using two-way analysis of variance. Firth's penalised likelihood method was applied in Cox regression models where standard maximum likelihood estimation failed to demonstrate convergence. Multivariate models were compared using likelihood ratio tests. Collinearity among covariates was evaluated through variance inflation factor analysis, covering all time-independent covariates, total cfDNA, absolute ddcfDNA, creatinine, WCC, rejection episodes, and detectable DSA. Relevant covariates were incorporated as time-dependent predictors to obviate correlation with outcomes.

Statistical analyses were conducted using R Statistical Software (v4.1.0; R Core Team 2021) with packages *car*, *coxphf*, *coxphw*, *ggplot2*, *gtsummary*, *rstatix*, *survival*, and *survminer*.

## 3 Results

### 3.1 Demographics

The study cohort consisted of 49 kidney transplant recipients with a median age of 55 years (IQR 42–62), over half of whom were male (27, 55%). Six participants from the original cohort were not included in the analysis as their follow-up occurred elsewhere. Nearly half of the participants had immunological primary kidney disease (23, 47%). The median follow-up duration for study participants was 6.3 years (IQR 6.0–7.2). Baseline patient and transplant demographic data is detailed in Table 1.

**Table 1:** Baseline demographic data.

Demographics and baseline characteristics		
Characteristic	*N* = 49	
	*n*	(%)
Age (years)^a^	55.1	(41.8, 61.6)
Sex		
Female	22	(45)
Male	27	(55)
Primary disease		
Genetic	7	(14)
Immune	23	(47)
Structural	10	(20)
Other	9	(18)
Follow up duration (years)^a^	6.3	(6.0, 7.2)
Transplant vintage (years)^a^	2.6	(0.5, 8.2)
Donor category		
Living	14	(29)
Deceased	34	(71)
Not documented	1	
Transplant number		
1	47	(96)
2	1	(2)
3	1	(2)
ABO compatible		
Yes	47	(100)
No	0	(0)
Not documented	2	
HLA mismatch		
≤3	32	(68)
≥4	15	(32)
Not documented	2	
Pre-existing DSA		
Present	11	(28)
Absent	29	(73)
Not documented	9	

DSA, donor-specific antibody; HLA, human leucocyte antigen.

^a^ Data presented as median (interquartile range).

Most participants (71%) had received transplants from deceased donors, with a median transplant vintage of 2.6 years (IQR 0.5–8.2) at the start of follow-up. Forty-seven participants had primary allografts *in situ*. All transplant donors were blood group compatible. Over two-thirds (68%) of participants had fewer than four donor HLA loci mismatches. Detectable DSAs were present in 11 participants (28%) prior to transplantation.

Maintenance immunosuppression at start of follow-up is presented in Supplementary Table S1. Slightly over half the participants (26, 53%) were maintained on standard triple immunosuppression (prednisolone, mycophenolate and tacrolimus). The mean number of immunosuppressive medications per participant was three (range 1–3).

#### 3.1.1 Histopathology

Primary histological diagnoses from the initial biopsy for each participant were classified according to the Banff schema (8), and are summarised in Table 2. Rejection was identified in one-third of biopsies (16, 33%), 22 biopsies (45%) were histologically normal, and the remaining 11 biopsies showed other pathologies.

**Table 2:** Primary histological diagnoses in original indication biopsies.

Initial indication biopsies		
Diagnosis	*N* = 49	
	*n*	(%)
Normal	22	(44.9)
Rejection	16	(32.7)
CNI toxicity	5	(10.2)
Primary disease	3	(6.12)
BK nephropathy	1	(2.0)
Interstitial nephritis	1	(2.0)
Other	1	(2.0)

CNI, calcineurin inhibitor.

The 16 initial biopsies indicating rejection comprised three acute AMR, two acute cellular-mediated rejection (CMR), two mixed acute AMR with acute CMR, six borderline CMR, two borderline AMR, and one mixed borderline AMR with borderline CMR. Acute AMR was therefore present in five biopsies, acute CMR in four biopsies, and mixed rejection in three biopsies. Data are presented in Supplementary Table S2.

During the follow-up period, 13 participants (27%) experienced at least one rejection episode (total of 17 episodes), including nine who had also experienced rejection in their initial biopsy. Details of subsequent rejection subtypes are provided in Supplementary Table S3. Across the study, 33 rejection episodes were recorded in 20 participants, ranging from one to three episodes per participant (mean 1.65, SD 0.75).

#### 3.1.2 Cell-free DNA results

The median total cfDNA concentration for the cohort was 1,751 cp/ml (IQR 986–4,020). The median absolute ddcfDNA concentration was 9 cp/ml (IQR 5–16), and the median fractional ddcfDNA was 0.44% (IQR 0.18–0.81).

Total cfDNA increased with age (*r* = 0.33, *P* = 0.02), while fractional ddcfDNA trended lower (*r* = −0.20, *P* = 0.16). Total cfDNA was lower with increasing transplant vintage (*r* = −0.31, *P* = 0.031), and fractional ddcfDNA trended higher (*r* = 0.21, *P* = 0.14). There was no correlation between absolute ddcfDNA and age or transplant vintage.

### 3.2 Patient and graft survival

During the study period, seven (14%) participants died, including four with a functioning graft and three with prior failed grafts (Figure 1). Participants who died were older at enrolment than those who survived (median age 64 years vs. 52 years, *P* < 0.01), and all had received transplants from deceased donors. For those who died, the median survival from study inclusion was 1,562 days (IQR 792–1,696). Known causes of death included infection (*n* = 3), cardiovascular disease (*n* = 1), dialysis withdrawal (*n* = 1), and other (*n* = 2) (Supplementary Table S4).

Seven participants (17%) experienced DCGL, with two subsequently undergoing graft nephrectomies. In total, 14 participants were classified as having AGL, including the four participants (29%) who died with functioning grafts. Among those with AGL, those with deceased donor transplants (8 participants) were older than the median cohort and significantly older than those with living donor transplants (5 participants; median age 65 years vs. 42 years, *P* < 0.01).

The overall median graft survival from study inclusion was 2,247 days (IQR 1,876–2,520). When restricting analysis to participants that survived, the median graft survival was 2,315 days (IQR 2,098–2,588) overall, 1,591 days (IQR 1,184–2,352) in those who lost grafts, and 2,324 days (IQR 2,208–2,574) in those with functioning grafts.

#### 3.2.1 Cell-free DNA according to outcome

Median values for each cfDNA parameter by outcome are provided in Supplementary Table S5, with boxplots for the primary outcomes shown in Supplementary Figure S1. No statistically significant differences in median cfDNA values were observed for any parameter or outcome.

Among those who died, there was a trend towards lower fractional ddcfDNA (0.26% vs. 0.52%, *P* = 0.07), compared to those who survived. In participants who died with functioning grafts, there was a trend towards higher absolute ddcfDNA (10 vs. 4 cp/ml, *P* = 0.05), compared to those who died following graft failure.

### 3.3 Survival analysis

#### 3.3.1 Univariate analysis

Optimal cfDNA cut points for the outcomes of death (total cfDNA 4,034 cp/ml, fractional ddcfDNA 0.67%, absolute ddcfDNA 16 cp/ml), AGL (total cfDNA 4,034 cp/ml, fractional ddcfDNA 0.09%, absolute ddcfDNA 21 cp/ml), and DCGL (total 1,265 cp/ml, fractional ddcfDNA 0.72%, absolute ddcfDNA 7 cp/ml) are presented in Supplementary Table S6. The table also presents median survival times for each subgroup stratified by cfDNA thresholds, and HRs derived from subgroup comparisons.

**Figure 2:**
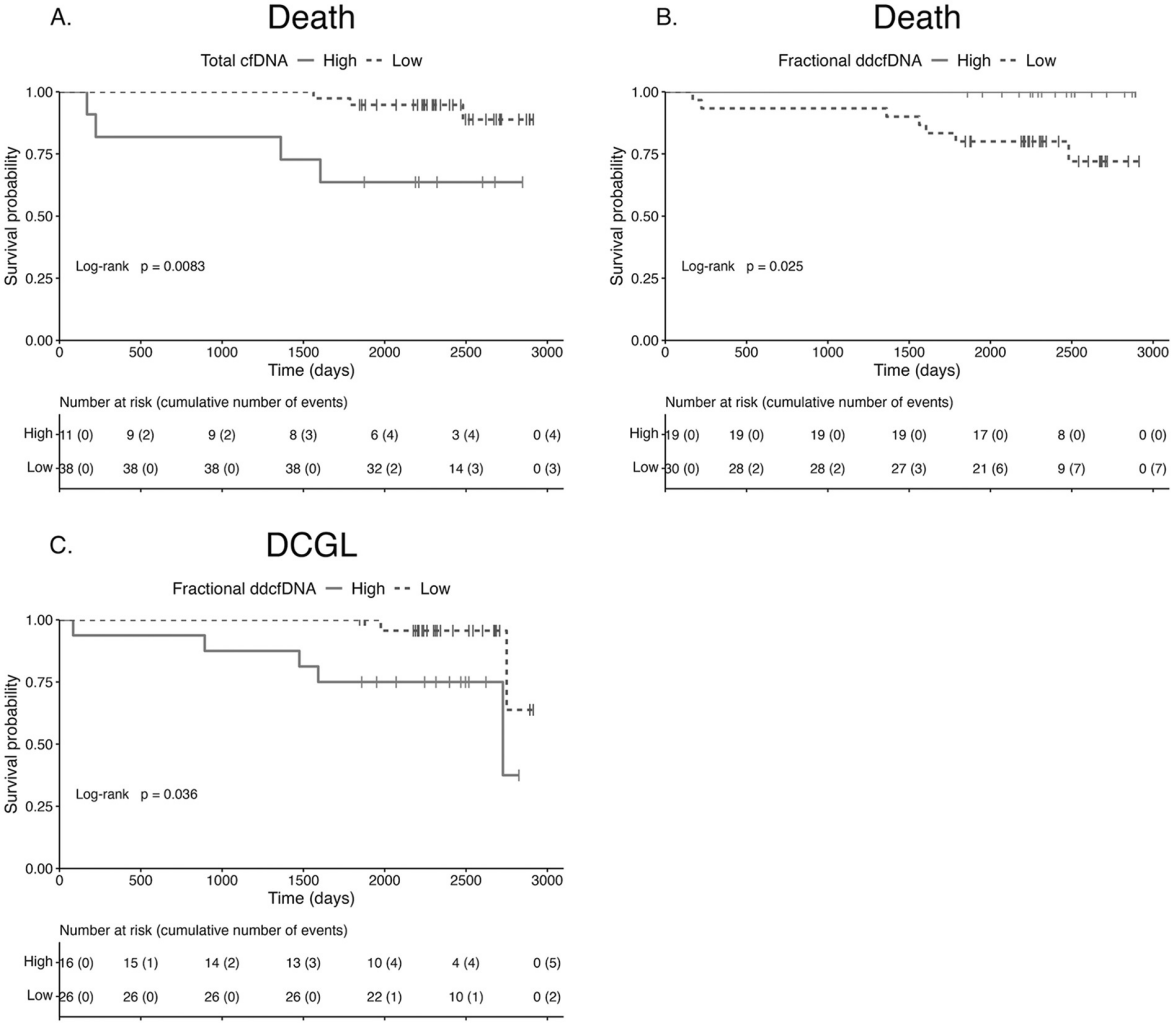
Survival according to cell-free DNA parameter. Kaplan–Meier survival curves for death stratified by total cfDNA **(A)** and fractional ddcfDNA **(B)**, and for DCGL stratified by fractional ddcfDNA **(C)**. The probability of death was significantly higher in participants with high total cfDNA (*P* = 0.008) and low fractional ddcfDNA (*P* = 0.025). The probability of DCGL was significantly higher in participants with high fractional ddcfDNA (*P* = 0.036). cfDNA, cell-free DNA; DCGL, death-censored graft loss; ddcfDNA, donor-derived cfDNA.

When stratifying the cohort by the relevant cfDNA threshold, the probability of death was significantly higher in participants with high total cfDNA (HR 5.94, 95% CI 1.40–25.13, *P* = 0.008) and in those with low fractional ddcfDNA (HR 10.85, 95% CI 1.32–1,409.19, *P* = 0.03). For DCGL, the probability was increased in participants with high fractional ddcfDNA (HR 4.93, 95% CI 1.12–21.72, *P* = 0.04). Corresponding Kaplan–Meier survival curves are shown in Figure 2.

For AGL, the probability was increased in participants with high total cfDNA, on weighted Cox regression analysis (average HR 2.62, Wald test *P* = 0.0497; generalised concordance probability 72.41%, 95% CI 50.03–87.31).

Stratification by absolute ddcfDNA did not yield statistically significant differences in survival probabilities.

##### 3.3.1.1 Time-independent covariates

On univariate analysis of time-independent covariates, only age was significantly associated with death (HR 1.21, 95% CI 1.06–1.37). Transplant vintage was significantly associated with both AGL (HR 1.09, 95% CI 1.03–1.16) and DCGL (HR 1.13, 95% CI 1.04–1.23). For AGL, there was a trend towards association with increasing age (HR 1.05, *P* = 0.08) and fewer than four HLA mismatches (HR 6.32, *P* = 0.08). For DCGL, there was a trend towards association with donor category (deceased donor HR 0.22, *P* = 0.07) and fewer than four HLA mismatches (penalised Cox regression HR 7.39, *P* = 0.07).

##### 3.3.1.2 Time-dependent covariates

Among the time-dependent covariates, increasing serum creatinine, urine PCR, and urine WCC were associated with all survival outcomes. Furthermore, increasing urine ACR was associated with AGL and DCGL, increasing urine RCC was associated with death, and decreasing blood lymphocyte count was associated with AGL.

The results of the univariate Cox models for death, AGL, and DCGL are presented in Supplementary Tables S7–S9, respectively.

#### 3.3.2 Multivariate analysis

Two multivariate Cox models were constructed for each primary outcome as described above, incorporating total cfDNA and fractional ddcfDNA. Absolute ddcfDNA was not included in multivariate models as it was not associated with primary outcomes on univariate analyses. Model 1 adjusted for pre-specified clinically relevant covariates, while Model 2 adjusted for covariates that were statistically significant on univariate analysis.

All multivariate models demonstrated statistically improved predictive performance for death, AGL, and DCGL compared to univariate prediction using total cfDNA or ddcfDNA. However, neither total cfDNA nor fractional ddcfDNA were independently predictive in any multivariate model. The results of these analyses are presented in Supplementary Tables S10-S12.

##### 3.3.2.1 Interaction effects

Given that all deaths occurred in older participants and that age positively correlated with total cfDNA, an interaction term combining age and total cfDNA was examined. On unadjusted analysis, death was predicted by the combination of increasing age and high total cfDNA (HR 1.36, 95% CI 1.03–1.80) and, to a lesser extent, by increasing age and low total cfDNA (HR 1.21, 95% CI 1.01–1.45). However, on multivariate analysis, inclusion of the interaction term did not improve prediction of death in either Model 1 or 2.

The interaction of age and total cfDNA was not independently predictive of AGL, despite a univariate trend towards increased allograft loss with increasing age.

The interaction of transplant vintage with cfDNA parameters was also examined, given the association between transplant vintage and AGL. Without adjustment, the combination of high total cfDNA and increasing transplant vintage was a stronger predictor for AGL (HR 1.54, 95% CI 1.14–2.09) compared to low total cfDNA and increasing transplant vintage (HR 1.14, 95% CI 1.06–1.22). Similarly, high fractional ddcfDNA combined with increasing transplant vintage predicted AGL (HR 1.12, 95% CI 1.05–1.19), while low fractional ddcfDNA combined with transplant vintage showed no significant association. On multivariate analysis, these interaction terms did not improve prediction of AGL in Model 1 or 2.

To explore the observed association between AGL and donor characteristics, an interaction term combining age and donor category was added to the multivariate models. This significantly improved prediction of AGL in Model 1 (*P* < 0.003). After adjusting for fractional ddcfDNA, HLA mismatch, pre-existing DSA, and rejection, AGL was independently predicted by high total cfDNA (HR 642, 95% CI 1.15–3.56 × 10^5^, *P* = 0.045), increasing transplant vintage (HR 1.2, 95% CI 1.01–1.43, *P* = 0.04), and the interaction of increasing age and donor category (global *P* = 0.021). These results are summarised in Table 3. Model 2 was not improved by addition of this interaction term.

**Table 3:** Multivariate Cox model for graft loss, adjusted for clinically relevant covariates, incorporating age and donor category interaction.

Graft loss			
Multivariate model (*n* = 9)			
Covariate	HR	95% CI	*P*
Total cfDNA (cp/ml)			
Low	–	–	
High	641.55	1.15, 3.56 × 10^5^	0.045
Fractional ddcfDNA (%)			
Low	–	–	
High	5.38	0.32, 90.50	0.24
Transplant vintage (per year)	1.20	1.01, 1.43	0.038
HLA mismatch^a^			
≥4	–	–	
≤3	1.12 × 10^8^	0.00, Inf	>0.99
Pre-existing DSA			
Absent	—	—	
Present	0.10	0.01, 1.74	0.11
Rejection^a^	0.00	0.00, Inf	>0.99
Donor category^b^			
Living	—	—	
Deceased	4.32	0.11, 176.91	0.44
Age (per year) + donor category			0.021
Age + living donor	0.70	0.51, 0.97	0.029
Age + deceased donor	1.09	0.82, 1.45	0.54

cfDNA, cell-free DNA; CI, confidence interval; DSA, donor specific antibody; HR, hazard ratio.

^a^ HLA mismatch and rejection did not converge on penalized Cox regression.

^b^ For recipients aged 55 years (median age of cohort).

## 4 Discussion

This study evaluated survival outcomes in a single-centre cohort of KTRs that was representative of the adult kidney transplant population (9, 10). The overall patient and allograft survival rates were consistent with local (9) and international (10–13) reports, but the proportion of death with graft function (29%) was lower than the national estimate of 50% (9).

Univariate analyses demonstrated that high total cfDNA and low fractional ddcfDNA predicted death over 6 years of follow-up. Elevated total cfDNA occurs in conditions such as critical illness, sepsis, and acute cardiovascular events, reflecting disease state at the time of measurement, due to its short half-life (∼2 h) (1). cfDNA can therefore be considered as a non-specific biomarker of health. However, its association with long-term mortality in this study suggests prognostic utility beyond immediate clinical status.

Similar associations between elevated cfDNA and mortality have been reported in sepsis (14, 15), acute kidney injury (AKI) (14, 16), trauma (17), haemodialysis (18), chronic obstructive pulmonary disease (19), and the general population (20). In SOTs, the prognostic validity of cfDNA has not been well characterised. Studies in heart transplant recipients have linked higher total cfDNA levels with cardiac arrest and mechanical circulatory support (21), and both short- and long-term mortality (22). A limited study of KTRs with COVID-19 infection suggested a correlation between cfDNA levels and mortality (20). The current study is the first to specifically evaluate the prognostic value of cfDNA in routine clinical practice in KTRs, demonstrating the long-term prognostic value of cfDNA measured at the time of indication biopsy as a predictor of mortality.

This is also the first study to report associations between cfDNA/ddcfDNA and allograft survival in KTRs. High total cfDNA predicted all-cause allograft loss, while high fractional ddcfDNA predicted DCGL. These findings align with reports linking ddcfDNA with estimated glomerular filtration rate changes and histological chronic injury in KTRs (2, 23). In heart and lung transplantation, higher ddcfDNA levels have been associated with allograft failure (24), cardiac allograft vasculopathy (22), rejection, and death (25), suggesting these analytes are not organ-specific predictors of adverse outcomes.

Mechanisms linking cfDNA to survival outcomes remain unclear. Elevated cfDNA levels are associated with acute illness and inflammation, which may serve as markers of general frailty or multimorbidity, contributing to higher mortality risk. In this study, the combination of high total cfDNA and low fractional ddcfDNA, particularly evident in the death with a functioning graft subgroup, may reflect a higher burden of immunosuppression. Over two-thirds of deaths in KTRs with functioning grafts are due to cardiovascular disease, infections, and malignancy—consequences of prolonged or excessive immunosuppression (9). This is further supported by the observed association between AGL and decreasing blood lymphocyte count, which can be attributed to both calcineurin inhibitor and mycophenolate mofetil exposure (26). Immunosuppression may increase total cfDNA through enhanced white cell turnover, while simultaneously lowering ddcfDNA by reducing allograft injury (26).

Lower tacrolimus exposure is associated with lower total cfDNA and higher fractional ddcfDNA in clinically stable KTRs (26). Over 1–5 years post-transplant, absolute ddcfDNA concentrations were stable, but total cfDNA steadily decreased, along with reducing tacrolimus concentrations (26). This correlation suggests that weaning of tacrolimus over time is associated with lower total cfDNA. Additional evidence supporting a relationship between tacrolimus and ddcfDNA includes high fractional ddcfDNA in liver transplant recipients with subtherapeutic tacrolimus levels in the first month after transplant (27), higher fractional and absolute ddcfDNA in KTRs with lower tacrolimus levels in the first year after transplant (28), higher fractional ddcfDNA in lung transplant recipients with “non-therapeutic” tacrolimus levels in the first two years after transplant (29), and higher fractional ddcfDNA in KTRs with highly variable tacrolimus levels in the first year after transplant (30). Longer follow up periods would permit investigation of relationships between total cfDNA/ddcfDNA and chronic sequelae of immunosuppression exposure.

An alternative explanation for the association between cfDNA and survival outcomes may involve the pathogenic effects of cfDNA itself. In addition to circulating freely, cfDNA is contained within extracellular vesicles and neutrophil extracellular traps, which can act as damage-associated molecular patterns (31). Studies in animal models and humans have shown that cfDNA activates pattern recognition receptors, triggering increased cytokine production, prolonging neutrophil viability (31), and contributing to tissue injury and cell death. Furthermore, cfDNA has been linked to disruptions in coagulation and fibrinolysis (31), as well as endothelial damage (20), suggesting a potential role in microvascular injury within kidney allografts. In murine models, cfDNA has been shown to provoke AKI through additional mechanisms, such as mitochondrial cfDNA-induced oxidative injury to kidney tubular cells in sepsis (32), and platelet activation leading to neutrophil extracellular trap formation in ischaemia reperfusion injury (33).

The predictive ability of cfDNA and ddcfDNA observed in univariate analyses in this study was not retained in all multivariate models, likely due to limited statistical power from the small cohort size, low event rates, and inclusion of multiple predictors. Nevertheless, an increasing body of literature supports the prognostic value of total cfDNA. Interestingly, other established predictors of death and allograft loss, such as recipient age, primary kidney disease aetiology, donor category, HLA mismatches, and rejection (12, 13, 34–36), were also not significant in this analysis. This finding may reflect model overfitting, which could obscure the effects of both cfDNA and these recognised risk factors. Larger cohort studies with more survival events are required to distinguish between underpowering, confounding, model overfitting, and statistical artefact as potential explanations for the observed discrepancies between univariate and multivariate results.

The multivariate model for AGL, incorporating the interaction term between recipient age and donor category, suggests a complex relationship between age, baseline allograft quality, allograft survival, and cfDNA concentration. While acknowledging the limitations of potential model overfitting, it is important to recognise the heterogeneity within transplant populations and the myriad factors influencing individual risk of adverse outcomes. No single biomarker can fully capture the complexity of prognostication. However, this study highlights the value of total cfDNA and ddcfDNA in enhancing predictions of allograft failure and mortality in a personalised manner. Additionally, the dynamic nature of cfDNA enables assessment of intervention effects, such as immunosuppression adjustments, through serial measurements, offering an advantage over static unmodifiable risk factors like donor category.

This study has several limitations. The small, single-centre cohort limits the development of robust prediction models. Only KTRs with clinical allograft dysfunction were included, precluding assessment of cfDNA prognostication in stable allograft function. The use of cut points for continuous numerical biomarkers reduces data granularity, potentially limiting interpretation in individual cases. However, cut points are often necessary for generalisation to larger cohorts and practical application in clinical settings, especially given variability among cfDNA assays from different manufacturers. Validation in larger, independent cohorts is needed to confirm these findings and to refine optimal cut point thresholds. Such studies would also address potential resubstitution bias due to the absence of a separate validation cohort. Lastly, most commercially available ddcfDNA assays do not quantify total cfDNA, requiring separate assays for measurement. This introduces additional costs, logistical challenges, and potential incompatibility with independently obtained ddcfDNA levels, complicating replication of this analysis.

This study demonstrates that absolute quantification of total cfDNA and fractional ddcfDNA (%) are predictive of mortality and allograft failure in adult KTRs with clinical allograft dysfunction over a 6-year follow-up period, whereas absolute quantification of ddcfDNA (cp/ml) is not predictive of survival outcomes. While ddcfDNA is an established biomarker of allograft rejection in SOT recipients, this study highlights the potential prognostic utility of total cfDNA and fractional ddcfDNA for long-term outcomes in KTRs. The association between elevated cfDNA and survival outcomes may reflect its pathogenic potential or serve as a marker of immunosuppression burden. Similar findings have been reported in heart and lung transplantation. We recognise the emergence of sophisticated multivariate prognostic tools that include other clinical and histological predictors of allograft failure (37, 38), but these tools are not universally accessible to transplant centres. Understanding the underlying pathogenic mechanisms and prognostic implications of cfDNA and ddcfDNA could improve personalised care for SOT recipients via a more widely available test, by facilitating titration of immunosuppression and screening for complications of over-immunosuppression. Further validation in larger cohorts is warranted to confirm these findings and optimise clinical application.

## Data Availability

The original contributions presented in the study are included in the article/Supplementary Material, further inquiries can be directed to the corresponding author.
