# Supplementary material for: Plasma total and donor-derived cell-free DNA predict survival in kidney transplant recipients
Source: Front Transplant. 2025 Sep 1;4:1624291. doi: 10.3389/frtra.2025.1624291 (PMC12434054; doi:10.3389/frtra.2025.1624291)
Supplement: Supplementary file 2 [file Table1.docx]

# SUPPLEMENTARY TABLES

| **Baseline immunosuppression (N = 49)** | |
| --- | --- |
| **Medication** | **n (%)** |
| Prednisolone | 41 (83.7) |
| Tacrolimus | 27 (55.1) |
| Tacrolimus XL | 5 (10.2) |
| Ciclosporin | 9 (18.4) |
| Mycophenolate mofetil | 25 (51.0) |
| Mycophenolic acid | 9 (18.4) |
| Azathioprine | 6 (12.2) |
| Everolimus | 1 (2.0) |
| Sirolimus | 2 (4.1) |
| Unknown | 3 (6.1) |

TABLE S1. Baseline maintenance immunosuppression. XL, extended release.

| **Initial rejection biopsies (N = 16)** | | | |
| --- | --- | --- | --- |
| **Rejection subtype** | | **Individual result**  **n (%)** | **Total cases**  **n (%)** |
| Acute AMR | | 3 (18.8) | 5 (31.2) |
| Acute CMR | | 2 (12.5) | 4 (25.0) |
| Borderline AMR | | 2 (12.5) | 3 (18.8) |
| Borderline CMR | | 6 (37.5) | 7 (43.8) |
| Mixed | aAMR + aCMR | 2 (12.5) | 3 (18.8) |
|  | bAMR + bCMR | 1 (6.25) |  |

TABLE S2. Rejection subtypes in original biopsies. aAMR, acute antibody-mediated rejection; aCMR, acute cellular-mediated rejection; bAMR, borderline AMR; bCMR, borderline CMR*.*

| **Subsequent rejection biopsies (N = 17)** | | | |
| --- | --- | --- | --- |
| **Rejection subtype** | | **Individual result**  **n (%)** | **Total cases**  **n (%)** |
| Acute AMR | | 2 (11.8) | 5 (29.4) |
| Acute CMR | | 3 (17.6) | 5 (29.4) |
| Chronic AMR | | 0 (0.0) | 2 (11.8) |
| Chronic CMR | | 1 (5.9) | 1 (5.9) |
| Borderline AMR | | 2 (11.8) | 3 (17.6) |
| Borderline CMR | | 4 (23.5) | 7 (41.2) |
| Mixed | aAMR + aCMR | 2 (11.8) | 5 (29.4) |
|  | cAMR + bCMR | 1 (5.9) |  |
|  | bAMR + bCMR | 1 (5.9) |  |
|  | aAMR + cAMR + bCMR | 1 (5.9) |  |

TABLE S3. Rejection subtypes in subsequent biopsies. aAMR, acute antibody-mediated rejection; aCMR, acute cellular-mediated rejection; bAMR, borderline AMR; bCMR, borderline CMR; cAMR, chronic AMR.

| **Deaths (N = 7)** | |
| --- | --- |
| **Cause of death** | **n (%)** |
| Infection  Septic shock +/- ischaemic bowel  Necrotising fasciitis of below knee amputation wound  Diabetic necrotic lower limb wounds | 3 (42.9) |
| Cardiovascular disease  Intracerebral haemorrhage | 1 (14.3) |
| Dialysis withdrawal | 1 (14.3) |
| Other  End stage fibrosing lung disease  Not reported | 2 (28.6) |

TABLE S4. Documented causes of death.

| **Median cfDNA level according to survival outcome** | | | | | | | | | |
| --- | --- | --- | --- | --- | --- | --- | --- | --- | --- |
| **Outcome** | **Result** | **Participants (n)** | **Total cfDNA (cp/mL)** | ***P*** | **Fractional ddcfDNA (%)** | ***P*** | **Absolute ddcfDNA (cp/mL)** | ***P*** |  |
| Death | Yes | 7 | 4046 | 0.19 | 0.26 | 0.07 | 6 | 0.28 |  |
|  | No | 42 | 1681 |  | 0.52 |  | 9 |  |  |
| AGL | Yes | 14 | 1534 | 0.96 | 0.46 | 0.85 | 9 | 0.97 |  |
|  | No | 35 | 1751 |  | 0.44 |  | 9 |  |  |
| DCGL | Yes | 7 | 1089 | 0.24 | 0.81 | 0.21 | 14 | 0.37 |  |
|  | No | 35 | 1751 |  | 0.44 |  | 9 |  |  |
| Failure | Yes | 10 | 1177 | 0.21 | 0.63 | 0.56 | 9 | 0.83 |  |
|  | No | 39 | 1851 |  | 0.37 |  | 9 |  |  |
| Death with a functioning graft | Yes | 4 | 6162 | 0.22 | 0.23 | 0.86 | 10 | 0.05 |  |
|  | No | 3 | 2225 |  | 0.26 |  | 4 |  |  |
| Nephrectomy | Yes | 2 | 1025 | 0.26 | 1.08 | 0.20 | 11 | 0.67 |  |
|  | No | 47 | 1803 |  | 0.40 |  | 9 |  |  |

TABLE S5. Median cfDNA results for each survival outcome. AGL, all graft loss; cfDNA, cell-free DNA; ddcfDNA, donor-derived cfDNA; DCGL, death-censored graft loss.

| **Survival analysis by cfDNA cut point** | | | | | |
| --- | --- | --- | --- | --- | --- |
| **Outcome** | **Parameter** | **Cut point** | **Median survival (days)** | **Hazard Ratio  (95% CI)** | **Log rank *P*** |
| Death | Total cfDNA | 4034 cp/mL |  |  |  |
|  | High |  | 2191 | **5.94 (1.40-25.13)** | **0.008** |
|  | Low |  | 2372 |  |  |
|  | Fract. ddcfDNA | 0.67% |  |  |  |
|  | High |  | 2470 |  |  |
|  | Low |  | 2282 | **10.85 (1.32-1409.19)*** | **0.03** |
|  | Absol. ddcfDNA | 16 cp/mL |  |  |  |
|  | High |  | 2624 |  |  |
|  | Low |  | 2261 | 5.58 (0.66-729.51)* | 0.11 |
| AGL | Total cfDNA | 4034 cp/mL |  |  |  |
|  | High |  | 2191 | **2.62 (1.001-6.88)**** | 0.12 |
|  | Low |  | 2282 |  |  |
|  | Fract. ddcfDNA | 0.09% |  |  |  |
|  | High |  | 2282 |  |  |
|  | Low |  | 2191 | 2.42 (0.67-8.74)** | 0.20 |
|  | Absol. ddcfDNA | 21 cp/mL |  |  |  |
|  | High |  | 2572 |  |  |
|  | Low |  | 2212 | 1.46 (0.28-7.56)** | 0.21 |
| DCGL | Total cfDNA | 1265 cp/mL |  |  |  |
|  | High |  | 2313 |  |  |
|  | Low |  | 2218 | 1.43 (0.27-7.71)** | 0.04 |
|  | Fract. ddcfDNA | 0.72% |  |  |  |
|  | High |  | 2282 | **4.93 (1.12-21.72)** | **0.04** |
|  | Low |  | 2240 |  |  |
|  | Absol. ddcfDNA | 7 cp/mL |  |  |  |
|  | High |  | 2324 | 3.89 (0.49-30.81) | 0.18 |
|  | Low |  | 2222 |  |  |

TABLE S6. Optimal cfDNA cut points and corresponding survival analysis results. Statistically significant findings are shown in bold. AGL, all graft loss; cfDNA, cell-free DNA; CI, confidence interval; DCGL, death-censored graft loss; ddcfDNA, donor-derived cfDNA.
*Penalized Cox regression.
**Weighted Cox regression, with weights truncated at the 97^th^ percentile.

| **Death** | | | | | |  |
| --- | --- | --- | --- | --- | --- | --- |
|  | **Obs (N)** | **Events (n)** | **HR** | **95% CI** | ***P*** |  |
| 1. **Time-independent variables** | | | | | | |
| Age | 49 | 7 | 1.21 | 1.06, 1.37 | 0.003 |  |
| Sex | 49 | 7 |  |  |  |  |
| Male |  |  | — | — |  |  |
| Female |  |  | 1.64 | 0.37, 7.33 | 0.52 |  |
| Primary disease | 49 | 7 |  |  |  |  |
| Other |  |  | — | — |  |  |
| Genetic |  |  | 0.00 | 0.00, Inf | >0.99 |  |
| Immune |  |  | 0.56 | 0.09, 3.37 | 0.53 |  |
| Structural |  |  | 0.90 | 0.13, 6.40 | 0.91 |  |
| Transplant vintage | 49 | 7 | 1.05 | 0.97, 1.14 | 0.19 |  |
| Donor age | | 47 | 6 | 1.02 | 0.97, 1.08 | 0.39 |
| Donor category | 48 | 6 |  |  |  |  |
| Living |  |  | — | — |  |  |
| Deceased |  |  | 3.47x10^8^ | 0.00, Inf | >0.99 |  |
| HLA mismatch | 47 | 6 |  |  |  |  |
| ≥4 |  |  | — | — |  |  |
| ≤3 |  |  | 2.47 | 0.29, 21.16 | 0.41 |  |
| Pre-existing DSA | 40 | 4 |  |  |  |  |
| Absent |  |  | — | — |  |  |
| Present |  |  | 0.89 | 0.09, 8.55 | 0.92 |  |
| 1. **Time-dependent variables**^1^ | | | | | | |
| Creatinine | | 534 | 7 | 1.06 | 1.01, 1.11 | 0.027 |
| Urine PCR | | 462 | 7 | 1.19 | 1.04, 1.36 | 0.010 |
| Urine ACR | | 300 | 4 | 1.55 | 0.98, 2.46 | 0.060 |
| Urine WCC | | 491 | 7 | 1.15 | 1.04, 1.26 | 0.004 |
| Urine RCC | | 491 | 7 | 1.16 | 1.04, 1.28 | 0.007 |
| WCC | | 505 | 7 | 0.99 | 0.77, 1.27 | 0.93 |
| Lymphocytes | | 505 | 7 | 0.44 | 0.13, 1.54 | 0.20 |
| Neutrophils | | 505 | 7 | 1.03 | 0.81, 1.30 | 0.83 |
| Tacrolimus level | | 415 | 5 | 1.06 | 0.68, 1.65 | 0.81 |
| ERL level | | 20 | 0 |  |  |  |
| CsA 2h level | | 103 | 3 | 1.49 | 0.71, 3.13 | 0.30 |
| SRL level | | 26 | 1 |  |  |  |
| CMV | | 551 | 7 | 0.00 | 0.00, Inf | >0.99 |
| BK | | 551 | 7 |  |  |  |
| Post transplant DSA | | 179 | 2 | 0.00 | 0.00, Inf | >0.99 |
| Rejection | | 551 | 7 | 0.00 | 0.00, Inf | >0.99 |
| *^1^* Creatinine per 10umol/L, urine PCR per 50mg/mmol, urine ACR per 50mg/mmol, urine WCC per 50 x10^6^/L, urine RCC per 50 x10^6^/L, WCC x10^9^/L, lymphocytes x10^9^/L, neutrophils x10^9^/L, tacrolimus level ug/L, ERL level ug/L, CsA per 100ng/mL, SRL level ug/L, number of CMV DNAemia episodes, number of BK viraemia episodes, detectable post transplant DSA, cumulative number of rejection episodes | | | | | | |

TABLE S7. Univariate Cox models for death. a) Time-independent variables. b) Time-dependent variables. Covariates that failed to converge in standard Cox models were not significant on penalized Cox regression. ACR, albumin-to-creatinine ratio; CI, confidence interval; CMV, cytomegalovirus; CsA, ciclosporin A; DSA, donor-specific antibody; ERL, everolimus; HR, hazard ratio; Obs, observations; PCR, protein-to-creatinine ratio; RCC, red cell count; SRL, sirolimus; WCC, white cell count.

| **AGL** | | | | | |  |
| --- | --- | --- | --- | --- | --- | --- |
|  | **Obs (N)** | **Events (n)** | **HR** | **95% CI** | ***P*** |  |
| 1. **Time-independent variables** | | | | | | |
| Age | 49 | 14 | 1.05 | 0.99, 1.10 | 0.080 |  |
| Sex | 49 | 14 |  |  |  |  |
| Male |  |  | — | — |  |  |
| Female |  |  | 1.61 | 0.55, 4.71 | 0.39 |  |
| Primary disease | 49 | 14 |  |  |  |  |
| Other |  |  | — | — |  |  |
| Genetic |  |  | 1.93 | 0.34, 11.03 | 0.46 |  |
| Immune |  |  | 0.65 | 0.12, 3.57 | 0.62 |  |
| Structural |  |  | 1.53 | 0.28, 8.51 | 0.62 |  |
| Transplant vintage | 49 | 14 | 1.09 | 1.03, 1.16 | 0.002 |  |
| Donor age | | 47 | 13 | 1.02 | 0.99, 1.06 | 0.23 |
| Donor category | 48 | 13 |  |  |  |  |
| Living |  |  | — | — |  |  |
| Deceased |  |  | 0.74 | 0.24, 2.28 | 0.60 |  |
| HLA mismatch | 47 | 13 |  |  |  |  |
| ≥4 |  |  | — | — |  |  |
| ≤3 |  |  | 6.32 | 0.82, 48.85 | 0.077 |  |
| Pre-existing DSA | 40 | 10 |  |  |  |  |
| Absent |  |  | — | — |  |  |
| Present |  |  | 1.11 | 0.29, 4.30 | 0.88 |  |
| 1. **Time-dependent variables**^1^ | | | | | | |
| Creatinine | | 490 | 14 | 1.21 | 1.13, 1.30 | <0.001 |
| Urine PCR | | 418 | 14 | 1.45 | 1.19, 1.75 | <0.001 |
| Urine ACR | | 271 | 6 | 2.12 | 1.28, 3.50 | 0.004 |
| Urine WCC | | 447 | 14 | 1.36 | 1.17, 1.58 | <0.001 |
| Urine RCC | | 447 | 14 | 1.07 | 0.95, 1.20 | 0.29 |
| WCC | | 461 | 14 | 0.93 | 0.74, 1.15 | 0.49 |
| Lymphocytes | | 461 | 14 | 0.34 | 0.13, 0.88 | 0.026 |
| Neutrophils | | 461 | 14 | 1.01 | 0.84, 1.21 | 0.90 |
| Tacrolimus level | | 377 | 11 | 1.01 | 0.75, 1.37 | 0.93 |
| ERL level | | 14 | 0 |  |  |  |
| CsA 2h level | | 92 | 5 | 0.95 | 0.63, 1.42 | 0.81 |
| SRL level | | 21 | 1 | 0.00 | 0.00, Inf | >0.99 |
| CMV | | 507 | 14 | 0.00 | 0.00, Inf | >0.99 |
| BK | | 507 | 14 | 0.00 | 0.00, Inf | >0.99 |
| Post transplant DSA | | 155 | 7 | 0.52 | 0.09, 3.16 | 0.48 |
| Rejection | | 507 | 14 | 0.00 | 0.00, Inf | >0.99 |
| *^1^* Creatinine per 10umol/L, urine PCR per 50mg/mmol, urine ACR per 50mg/mmol, urine WCC per 50 x10^6^/L, urine RCC per 50 x10^6^/L, WCC x10^9^/L, lymphocytes x10^9^/L, neutrophils x10^9^/L, tacrolimus level ug/L, ERL level ug/L, CsA per 100ng/mL, SRL level ug/L, number of CMV DNAemia episodes, number of BK viraemia episodes, detectable post transplant DSA, cumulative number of rejection episodes | | | | | | |

TABLE S8. Univariate Cox models for all graft loss (AGL). a) Time-independent variables. b) Time-dependent variables. Covariates that failed to converge in standard Cox models were not significant on penalized Cox regression. ACR, albumin-to-creatinine ratio; CI, confidence interval; CMV, cytomegalovirus; CsA, ciclosporin A; DSA, donor-specific antibody; ERL, everolimus; HR, hazard ratio; Obs, observations; PCR, protein-to-creatinine ratio; RCC, red cell count; SRL, sirolimus; WCC, white cell count.

| **DCGL** | | | | | |  |
| --- | --- | --- | --- | --- | --- | --- |
|  | **Obs (N)** | **Events (n)** | **HR** | **95% CI** | ***P*** |  |
| 1. **Time-independent variables** | | | | | | |
| Age | 42 | 7 | 0.99 | 0.93, 1.05 | 0.74 |  |
| Sex | 42 | 7 |  |  |  |  |
| Male |  |  | — | — |  |  |
| Female |  |  | 1.47 | 0.31, 6.88 | 0.62 |  |
| Primary disease | 42 | 7 |  |  |  |  |
| Other |  |  | — | — |  |  |
| Genetic |  |  | 4.96x10^8^ | 0.00, Inf | >0.99 |  |
| Immune |  |  | 4.81x10^7^ | 0.00, Inf | >0.99 |  |
| Structural |  |  | 2.31x10^8^ | 0.00, Inf | >0.99 |  |
| Transplant vintage | 42 | 7 | 1.13 | 1.04, 1.23 | 0.003 |  |
| Donor age | | 41 | 7 | 1.02 | 0.98, 1.06 | 0.41 |
| Donor category | 42 | 7 |  |  |  |  |
| Living |  |  | — | — |  |  |
| Deceased |  |  | 0.22 | 0.04, 1.12 | 0.068 |  |
| HLA mismatch | 41 | 7 |  |  |  |  |
| ≥4 |  |  | — | — |  |  |
| ≤3 |  |  | 3.53x10^8^ | 0.00, Inf | >0.99 |  |
| Pre-existing DSA | 36 | 6 |  |  |  |  |
| Absent |  |  | —- | — |  |  |
| Present |  |  | 1.36 | 0.25, 7.46 | 0.73 |  |
| 1. **Time-dependent variables**^1^ | | | | | | |
| Creatinine | | 433 | 7 | 1.25 | 1.10, 1.43 | <0.001 |
| Urine PCR | | 368 | 7 | 1.66 | 1.21, 2.29 | 0.002 |
| Urine ACR | | 253 | 3 | 2.69 | 1.12, 6.44 | 0.027 |
| Urine WCC | | 394 | 7 | 1.27 | 1.04, 1.54 | 0.019 |
| Urine RCC | | 394 | 7 | 0.85 | 0.39, 1.89 | 0.70 |
| WCC | | 404 | 7 | 0.77 | 0.52, 1.12 | 0.17 |
| Lymphocytes | | 404 | 7 | 0.22 | 0.05, 1.02 | 0.052 |
| Neutrophils | | 404 | 7 | 0.91 | 0.64, 1.30 | 0.61 |
| Tacrolimus level | | 338 | 6 | 0.77 | 0.49, 1.21 | 0.26 |
| ERL level | | 14 | 0 |  |  |  |
| CsA 2h level | | 66 | 2 | 0.00 | 0.00, Inf | >0.99 |
| SRL level | | 11 | 0 |  |  |  |
| CMV | | 450 | 7 | 0.00 | 0.00, Inf | >0.99 |
| BK | | 450 | 7 | 0.00 | 0.00, Inf | >0.99 |
| Post transplant DSA | | 142 | 5 | 1.26 | 0.11, 13.9 | 0.85 |
| Rejection | | 450 | 7 | 0.00 | 0.00, Inf | >0.99 |
| *^1^* Creatinine per 10umol/L, urine PCR per 50mg/mmol, urine ACR per 50mg/mmol, urine WCC per 50 x10^6/L, urine RCC per 50 x10^6/L, WCC x10^9^/L, lymphocytes x10^9^/L, neutrophils x10^9^/L, tacrolimus level ug/L, ERL level ug/L, CsA per 100ng/mL, SRL level ug/L, number of CMV DNAemia episodes, number of BK viraemia episodes, detectable post transplant DSA, cumulative number of rejection episodes | | | | | | |

TABLE S9. Univariate Cox models for death-censored graft loss (DCGL). a) Time-independent variables. b) Time-dependent variables. Covariates that failed to converge in standard Cox models were not significant on penalized Cox regression. ACR, albumin-to-creatinine ratio; CI, confidence interval; CMV, cytomegalovirus; CsA, ciclosporin A; DSA, donor-specific antibody; ERL, everolimus; HR, hazard ratio; Obs, observations; PCR, protein-to-creatinine ratio; RCC, red cell count; SRL, sirolimus; WCC, white cell count.

| **Death** | | | |
| --- | --- | --- | --- |
| 1. **Model 1** (n = 5) | | | |
| **Variable** | **HR**^1^ | **95% CI**^2^ | ***P*** |
| Total cfDNA (cp/mL) |  |  |  |
| Low | — | — |  |
| High | 21.03 | 0.16, 2.83x10^3^ | 0.22 |
| Fractional ddcfDNA (%) |  |  |  |
| Low | — | — |  |
| High | 0.00 | 0.00, Inf | >0.99 |
| Age (per year) | 1.24 | 0.94, 1.63 | 0.13 |
| Transplant vintage (per year) | 1.18 | 0.84, 1.67 | 0.34 |
| Donor category |  |  |  |
| Living | — | — |  |
| Deceased | 2.42x10^9^ | 0.00, Inf | >0.99 |
| HLA mismatch |  |  |  |
| ≥4 | — | — |  |
| ≤3 | 4.20 | 0.09, 195.63 | 0.46 |
| Rejection | 3.27 | 0.00, Inf | >0.99 |
| 1. **Model 2** (n = 7) | | | |
| **Variable** | **HR**^1^ | **95% CI**^2^ | ***P*** |
| Total cfDNA (cp/mL) |  |  |  |
| Low | — | — |  |
| High | 6.62 | 0.39, 111.77 | 0.19 |
| Fractional ddcfDNA (%) |  |  |  |
| Low | — | — |  |
| High | 0.00 | 0.00, Inf | >0.99 |
| Age (per year) | 1.26 | 0.99, 1.60 | 0.056 |
| Creatinine^3^ | 1.09 | 0.94, 1.25 | 0.24 |
| Urine PCR^3^ | 1.17 | 0.78, 1.75 | 0.45 |
| Urine WCC^3^ | 0.94 | 0.68, 1.29 | 0.69 |
| Urine RCC^3^ | 1.53 | 0.90, 2.60 | 0.12 |
| *^1^* HR = Hazard Ratio | | | |
| *^2^* CI = Confidence Interval  ^3^ Creatinine per 10umol/L, urine PCR per 50mg/mmol, urine WCC per 50 x10^6/L, urine RCC per 50 x10^6/L | | | |

TABLE S10. Multivariate Cox models for death. a) Model 1: adjusted for clinically relevant covariates. b) Model 2: adjusted for covariates with statistical significance on univariate analysis (urine ACR omitted due to missing data). Graft fraction, donor category, and rejection were not significant on penalized Cox regression. cfDNA, cell-free DNA; PCR, protein-to-creatinine ratio; RCC, red cell count; WCC, white cell count.

| **AGL** | | | |
| --- | --- | --- | --- |
| 1. **Model 1** (n = 9) | | | |
| **Variable** | **HR**^1^ | **95% CI**^2^ | ***P*** |
| Total cfDNA (cp/mL) |  |  |  |
| Low | — | — |  |
| High | 3.04 | 0.11, 81.52 | 0.51 |
| Fractional ddcfDNA (%) |  |  |  |
| Low | — | — |  |
| High | 0.16 | 0.02, 1.33 | 0.09 |
| Age (per year) | 0.99 | 0.88, 1.12 | 0.92 |
| Transplant vintage (per year) | 1.15 | 1.01, 1.30 | 0.035 |
| Donor category |  |  |  |
| Living | — | — |  |
| Deceased | 0.75 | 0.11, 5.17 | 0.77 |
| HLA mismatch |  |  |  |
| ≥4 | — | — |  |
| ≤3 | 3.42x10^8^ | 0.00, Inf | >0.99 |
| Pre-existing DSA |  |  |  |
| Absent | — | — |  |
| Present | 2.10 | 0.27, 16.37 | 0.48 |
| Rejection | 0.00 | 0.00, Inf | >0.99 |
| 1. **Model 2** (n = 13) | | | |
| **Variable** | **HR**^1^ | **95% CI**^2^ | ***P*** |
| Total cfDNA (cp/mL) |  |  |  |
| Low | — | — |  |
| High | 17.01 | 0.19, 1.49x10^3^ | 0.21 |
| Fractional ddcfDNA (%) |  |  |  |
| Low | — | — |  |
| High | 1.22 | 0.10, 14.30 | 0.88 |
| Age (per year) | 0.99 | 0.85, 1.14 | 0.85 |
| Transplant vintage (per year) | 1.10 | 0.89, 1.34 | 0.38 |
| HLA mismatch |  |  |  |
| ≥4 | — | — |  |
| ≤3 | 0.64 | 0.02, 19.77 | 0.80 |
| Creatinine^3^ | 1.17 | 1.05, 1.30 | 0.005 |
| Urine PCR^3^ | 1.22 | 0.97, 1.52 | 0.086 |
| Urine WCC^3^ | 1.16 | 0.92, 1.46 | 0.21 |
| Lymphocytes^3^ | 0.31 | 0.05, 1.96 | 0.22 |
| *^1^* HR = Hazard Ratio | | | |
| *^2^* CI = Confidence Interval  ^3^ Creatinine per 10umol/L, urine PCR per 50mg/mmol, urine WCC per 50 x10^6/L, lymphocytes x10^9/L | | | |

TABLE S11. Multivariate Cox models for all graft loss (AGL). a) Model 1: adjusted for clinically relevant covariates. b) Model 2: adjusted for covariates with statistical significance on univariate analysis (urine ACR omitted due to missing data). HLA mismatch and rejection were not significant on penalized Cox regression. cfDNA, cell-free DNA; DSA, donor specific antibody; PCR, protein-to-creatinine ratio; WCC, white cell count.

| **DCGL** | | | |
| --- | --- | --- | --- |
| 1. **Model 1** (n = 6) | | | |
| **Variable** | **HR**^1^ | **95% CI**^2^ | ***P*** |
| Total cfDNA (cp/mL) |  |  |  |
| Low | — | — |  |
| High | 1.82x10^41^ | 0.00, Inf | >0.99 |
| Fractional ddcfDNA (%) |  |  |  |
| Low | — | — |  |
| High | 6.51x10^19^ | 0.00, Inf | >0.99 |
| Age (per year) | 0.00 | 0.00, 8.65x10^213^ | 0.97 |
| Transplant vintage (per year) | 9.12x10^12^ | 0.00, Inf | 0.97 |
| Donor category |  |  |  |
| Living | — | — |  |
| Deceased | 0.00 | 0.00, Inf | 0.97 |
| HLA mismatch |  |  |  |
| ≥4 | — | — |  |
| ≤3 | 6.13x10^99^ | 0.00, Inf | >0.99 |
| Pre-existing DSA |  |  |  |
| Absent | — | — |  |
| Present | 0.00 | 0.00, Inf | >0.99 |
| Rejection | 2.63x10^42^ | 0.00, Inf | >0.99 |
| 1. **Model 2** (n = 7) | | | |
| **Variable** | **HR**^1^ | **95% CI**^2^ | ***P*** |
| Total cfDNA (cp/mL) |  |  |  |
| Low | — | — |  |
| High | 0.00 | 0.00, Inf | 0.99 |
| Fractional ddcfDNA (%) |  |  |  |
| Low | — | — |  |
| High | 286.67 | 0.00, 1.16x10^225^ | 0.98 |
| Transplant vintage (per year) | 1.15 | 0.00, 2.23x10^11^ | >0.99 |
| Donor category |  |  |  |
| Living | — | — |  |
| Deceased | 106.16 | 0.00, 9.09x10^268^ | 0.99 |
| HLA mismatch |  |  |  |
| ≥4 | — | — |  |
| ≤3 | 287.98 | 0.00, 5.55x10^281^ | 0.99 |
| Creatinine^3^ | 2.45 | 0.00, 5.34x10^16^ | 0.96 |
| Urine PCR^3^ | 1.69 | 0.00, 1.21x10^6^ | 0.94 |
| Urine WCC^3^ | 2.22 | 0.00, 4.47x10^30^ | 0.98 |
| Lymphocytes^3^ | 291.63 | 0.00, 4.01x10^89^ | 0.96 |
| *^1^* HR = Hazard Ratio | | | |
| *^2^* CI = Confidence Interval  ^3^ Creatinine per 10umol/L, urine PCR per 50mg/mmol, urine WCC per 50 x10^6/L, lymphocytes x10^9/L | | | |

TABLE S12. Multivariate Cox models for death-censored graft loss (DCGL). a) Model 1: adjusted for clinically relevant covariates; penalized Cox regression: living donor HR 149 (*P* = 0.03), transplant vintage HR 1.49 (*P* = 0.08); total cfDNA, graft fraction, age, HLA mismatch, pre-existing DSA and rejection not significant. b) Model 2: adjusted for covariates with statistical significance on univariate analysis (urine ACR omitted due to missing data). cfDNA, cell-free DNA; DSA, donor specific antibody; PCR, protein-to-creatinine ratio; WCC, white cell count.
